# Patterns and correlates of intention to use contraceptives among fecund sexually active women in developing countries

**DOI:** 10.1080/16549716.2023.2255043

**Published:** 2023-09-08

**Authors:** Clifford Odimegwu, Million Phiri, Talent Tapera, Simona Simona

**Affiliations:** a Demography and Population Studies Programme, Schools of Public Health and Social Sciences, University of the Witwatersrand, Johannesburg, South Africa; b Department of Population Studies, School of Humanities and Social Sciences, University of Zambia, Lusaka, Zambia; c Department of Social Work and Sociology, School of Humanities and Social Sciences, University of Zambia, Lusaka, Zambia

**Keywords:** Women, contraceptive use intention, reproductive health, family planning, global health

## Abstract

**Background:**

Understanding a woman’s future contraceptive needs and enhancing her chances of putting those needs into action depend heavily on her intentions to use contraceptive methods. However, there is little information about global perspectives of intention to utilise contraceptives among fecund sexually active women.

**Objectives:**

This study examines the patterns and determinants of contraception intention of fecund sexually active women.

**Methods:**

The most recent Demographic and Health Surveys (DHS) from 59 countries were used for secondary data analysis. The DHSs applied a cross-sectional survey design to collect data from women between the ages of 15 and 49. The study comprises a sample of 697,590 fecund sexually active women in the reproductive ages. The desire to utilise contraceptive methods was examined using a multivariable binary logistic regression analysis. All analyses were weighted to allow for a complex survey design.

**Results:**

A pooled prevalence of intention to utilise contraception was 42.8% (95% CI: 42.5, 43.1) at the global level. Eastern and Southern Europe had the lowest prevalence, 17.3% (95% CI: 16.4, 18.2), and the highest prevalence was observed in countries from Latin America and the Caribbean, 68.0% (95% CI: 67.5, 69.9). Attaining secondary-level education (adjusted odds ratio (aOR) = 1.68; 95% CI: 1.62–1.72) or higher (aOR = 1.71; 95% CI: 1.63–1.80), working (aOR = 1.21; 95% CI: 1.18–1.24), experience of a pregnancy loss (aOR = 1.06; 95% CI: 1.03–1.09), or being exposed to media family planning messages (aOR = 1.51; 95% CI: 1.48–1.55) were factors associated with an increased likelihood of intent to use contraceptives.

**Conclusions:**

The study has established that contraceptive use intention was low in many developing countries. Education, age, employment status, fertility preference, and exposure to family planning messages influenced contraceptive use intention. Health policy-makers ought to consider these factors when designing sexual and reproductive health strategies in developing countries.

## Background

Utilising modern contraceptive methods reduces the likelihood of unplanned pregnancies and lowers maternal mortality and infant death [1–3]. Therefore, intent to utilise contraception among reproductive-aged women is important, because it has the potential to help women achieve their future reproductive needs. Given that it may be able to predict women’s health-seeking behaviours, the willingness to use contraceptive methods may constitute a helpful indicator when determining the outcomes of maternal health interventions [4]. Thus, understanding the drivers of women’s future contraceptive behaviour is imperative for informing family planning (FP) policy and programme design aimed at meeting women’s future sexual and reproductive health needs and increasing the chances of converting their intentions into action [5,6]. The demand for FP services is informed by information about the intention to use contraceptives [7,8].

In most developing countries, like those in Sub-Saharan Africa and Asia, unintended pregnancies, high fertility, and abortion are still public health challenges for many women of childbearing age [9–13]. It is therefore important that women’s access to sexual and reproductive health information and services ought to be recognised as a basic human right [6,14,15]. The health behaviour model explains that a person’s intention to perform a health behaviour is driven by the perceived risks, costs, and benefits of the action [16,17]. Use of FP is considered to be one of the key strategies to control family size and reduce the prevalence of unintended pregnancies in developing nations [17–19]. Even though the health benefits of using contraceptive methods among women are widely known, the prevalence of sexually active women currently using or intending to use contraceptives in the future is low in many developing countries [4,15,20,21].

Through the Family Planning Initiative (FP2030), the international community has agreed in principle regarding strategies to improve FP use by promoting access to information and contraceptive commodities to achieve the targets of the Sustainable Development Goal 3. These include reducing exposure to unwanted pregnancies [22], limiting births [1,23,24], reducing unsafe abortion [11,25], and decreasing newborn and maternal death rates [26]. According to the United Nations, in 2019, over 1.1 billion women of childbearing age needed contraceptive methods. However, approximately 270 million of these women’s needs for contraceptive methods went unfulfilled [27]. Additionally, the 2017 World Family Planning Report found that although women are generally satisfied with FP at a rate of about 78%, the percentage of its usage in developing nations is still low at 56%, due to several barriers [28]. The aim of the current study was to examine the patterns and correlates of contraceptive use intention among fecund sexually active women in developing countries. The findings could potentially inform the need for strengthening existing sexual and reproductive health policies and FP strategies aimed at accelerating global maternal health outcomes.

## Methods and data

### Data source

The most recent Demographic and Health Surveys (DHS) conducted in 59 countries between January 2007 and December 2021 were employed in this study. The DHS programme is a nationally representative household survey designed to collect various demographic, health, women empowerment, and domestic violence indicators in most developing countries. The surveys are usually implemented by the national statistics agencies, with support from global partners, including the ICF International and United States Agency for International Development. DHS use four standardised questionnaires to collect country-level data, including a household questionnaire, a woman’s questionnaire, a man’s questionnaire, and a biomarker questionnaire. A detailed description of the methods used in these surveys is included in the respective country's survey reports [29]. The individual women’s recode file, which comprises information of all females in the age group 15–49 who were interviewed, was used in this investigation. DHS website (https://dhsprogram.com/) can be used by the public to access datasets for all the countries that were used in this study [29]. Because our study focused on contraceptive intentions, our analysis was limited to fecund sexually active women, who were non-contraceptive users at the time of interview. The respective samples of women included for each country are described in Table 1.

**Table 1.** Description of information of DHS survey years and analysis sample by country and region (*N* = 697,590).

Country	Survey year	Women interviewed	Sample	Region
Angola	2015–16	14,379	10,912	Africa
Benin	2015	15,928	11,202	Africa
Burundi	2010	17,269	8,807	Africa
Burkina Faso	2010	17,087	11,822	Africa
Chad	2014–15	17,719	13,565	Africa
Cameroon	2018	14,677	8,093	Africa
Congo	2011–12	10,819	5,023	Africa
Congo DR	2013–14	18,827	12,809	Africa
Comoros	2012	5,329	2,823	Africa
Cote d’Ivoire	2011–12	10,060	7,070	Africa
Egypt	2014	21,762	9,666	Africa
Ethiopia	2016	15,683	8,385	Africa
Gambia	2019	11,865	7,140	Africa
Gabon	2012	8,422	5,007	Africa
Guinea	2018	10,874	7,482	Africa
Ghana	2014	9,396	5,824	Africa
Kenya	2014	31,079	6,630	Africa
Liberia	2019–20	8,965	4,938	Africa
Malawi	2015–16	24,562	10,087	Africa
Madagascar	2021	18,869	9,028	Africa
Mali	2018	10,519	7,296	Africa
Mauritania	2019–20	15,714	13,831	Africa
Mozambique	2011	13,745	10,059	Africa
Namibia	2013	10,018	3,312	Africa
Nigeria	2018	41,821	28,484	Africa
Rwanda	2019–20	14,634	4,791	Africa
Senegal	2019	8,649	4,786	Africa
Sierra Leone	2019	15,574	9,807	Africa
South Africa	2016	8,614	3,361	Africa
Tanzania	2015–16	13,266	7,157	Africa
Togo	2013–14	9,480	6,297	Africa
Uganda	2016	18,506	10,098	Africa
Zimbabwe	2015	9,955	3,185	Africa
Zambia	2018	13,683	6,883	Africa
Afghanistan	2011	29,461	20,764	Asia
Armenia	2015	6,116	1,691	Asia
Bangladesh	2014	20,127	7,369	Asia
Cambodia	2014	1,7578	5,957	Asia
Indonesia	2012	49,627	15,670	Asia
India	2016	699,686	240,968	Asia
Jordan	2017	14,689	7,034	Asia
Krygyz	2012	8,208	3,450	Asia
Myanmar	2014	12,885	4,560	Asia
Nepal	2016	12,862	4,703	Asia
Pakistan	2013	15,068	9,827	Asia
Philippines	2013	25,074	8,810	Asia
Tajikistan	2017	10,718	5,248	Asia
Timor-Leste	2010	12,607	5,906	Asia
Turkey	2018	9,746	4,330	Asia
Yemen	2013	25,434	10,732	Asia
Colombia	2015	38,718	8,385	Latin America and the Caribbean
Dominican	2013	9,372	2,612	Latin America and the Caribbean
Guatemala	2015	25,914	8,642	Latin America and the Caribbean
Honduras	2012	22,757	7,037	Latin America and the Caribbean
Haiti	2018	15,513	8,122	Latin America and the Caribbean
Albania	2017	15,000	4,800	Eastern and Southern Europe
Azerbaijan	2006	8,444	2,760	Eastern and Southern Europe
Moldova	2005	16,798	10,474	Eastern and Southern Europe
Ukraine	2007	6,841	2,079	Eastern and Southern Europe

### Study measures

#### Outcome measure

The outcome variable for this study was ‘contraceptive use intention’, focusing on sexually active women who either had intention to utilise contraception later or had no intention to use. Most studies on contraception globally indicate that improving the uptake of modern contraceptives remains an issue requiring urgent attention in many developing countries [30–33]. In the DHS, all women who were engaging in sexual activity but were not pregnant were questioned about whether they used any form of contraception to avoid getting pregnant. Based on this question, we restricted the analysis to only women who indicated they wanted to use contraceptive methods later or had no intention to use them. To perform a multivariable binary logistic regression model, the outcome variable was categorised as follows: one wanted to use it later or had no intention to use.

#### Independent variables

Based on existing literature, factors associated with contraception use in different parts of the world were selected [9,34–39]. Predictor variables were classified as follows: age of a woman (15–24, 25–34, and 35–49), education attainment (no education, primary, secondary, or higher), household wealth quintiles (poor, middle, or rich), area of residence (urban or rural), working status (not working or working), number of children ever had (zero, one to two, three to four, five, or more), number of under-5-year-olds in the household (0, 1, 2+), experience of a pregnancy loss (yes, no), desire for more children (no, yes), distance to the health facility (classified as a big challenge or not a big challenge), ownership of radio (yes, no), and exposure to media FP messages (yes, no).

#### Statistical analysis

The analysis took into consideration survey design and post-stratification weights, as defined in DHS datasets. A sample weight equalisation process was performed to give equal weight to each survey, such that if one survey had a large sample, it did not predominate the pooled results. Statistical analysis was delineated by region for comparison purposes and was done in three stages. First, univariate analysis was performed to describe the variations in the prevalence of intention to use contraception among women in each country and region. Then, bivariate analysis was performed through cross-tabulation to measure associations between independent correlates with the outcome measure using Pearson’s chi-square test. Last, binary multivariate logistic regression analysis models were performed for all identified independent correlates. Adjusted odds ratios (aOR) with corresponding p-values are reported. All covariates that showed a probability value of less than 0.20 at bivariate analysis were included in multivariable analysis. The variance inflation factor was applied to check multicollinearity among independent variables. All values were less than 5. Therefore, there was no multicollinearity between independent variables since variance inflation factor values ranged from 1.06 to 2.25, with a mean variance inflation factor of 1.36. Statistical analyses were done in Stata software version 17 with 95% confidence interval

## Results

### Prevalence of contraceptive use intention

**Figure 1.**
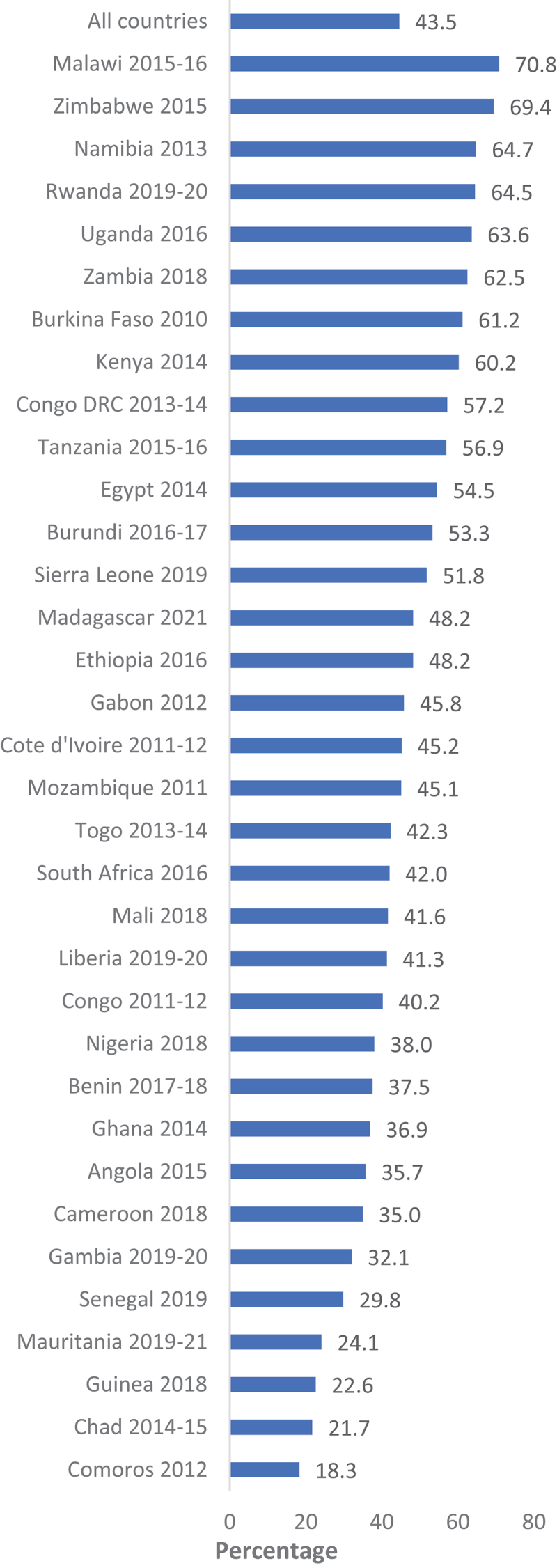
Prevalence of contraception use intention in Africa.

**Figure 2.**
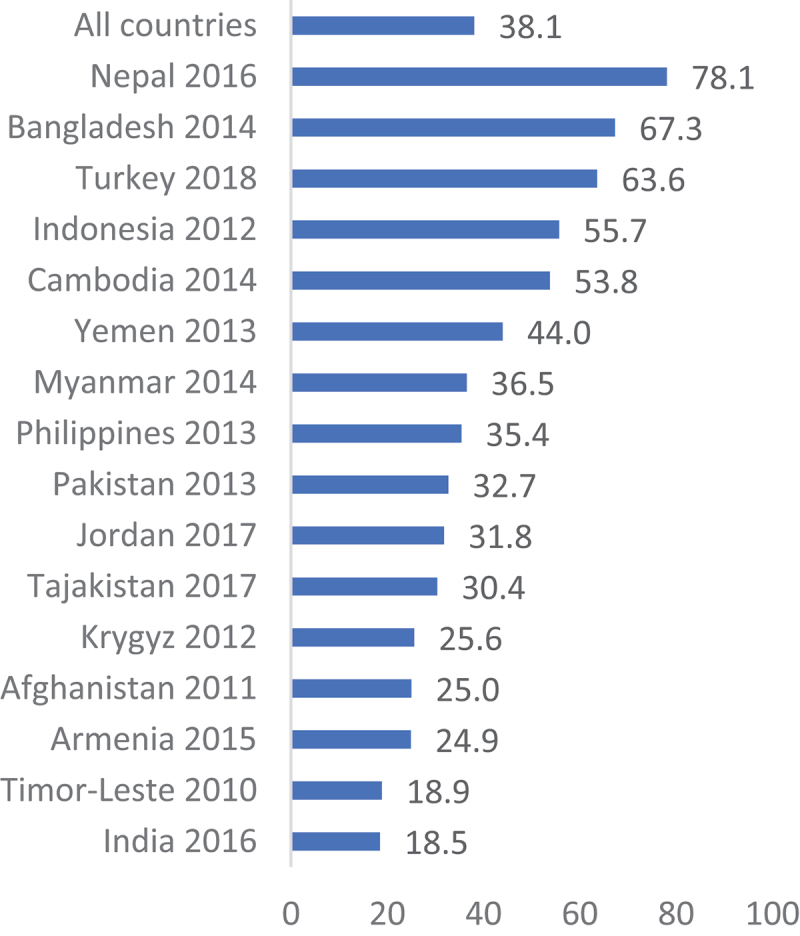
Prevalence of contraception use intention in Asia.

**Figure 3.**
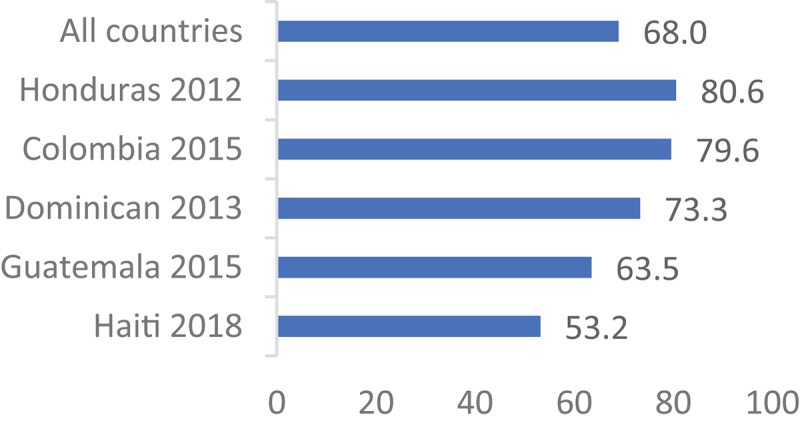
Prevalence of contraception use intention in Latin American and the Caribbean.

**Figure 4.**
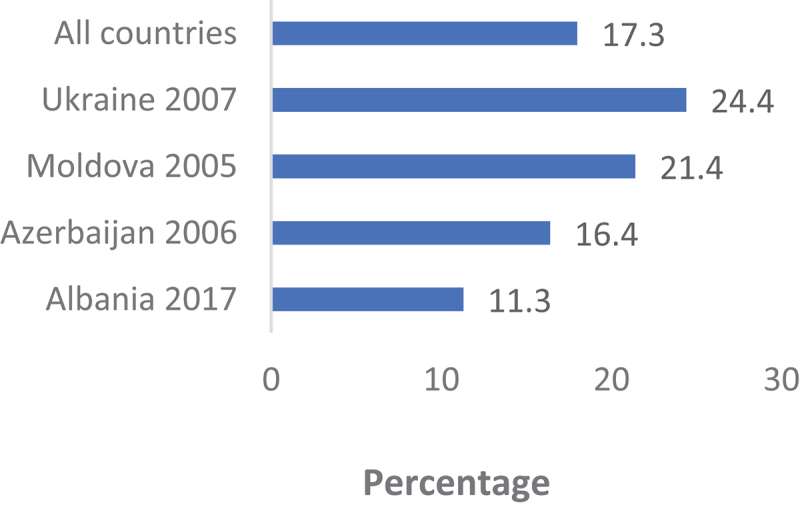
Prevalence of contraception use intention in Eastern and Southern Europe.

Figures 1–4 show a country-level prevalence of intention to utilise contraceptive methods among females in the childbearing age group in four regions. The overall prevalence of intention to utilise contraceptives for the 34 African countries included was 43.5% (95% CI: 43.1, 43.9). In Asia, the prevalence was 38.1% (95% CI: 37.6, 38.8) for 16 countries. Figure 3 shows that 68.0% (95% CI: 67.5, 69.9) of the women in Latin America and the Caribbean had the intention to use contraception. Furthermore, Eastern and Southern Europe had the lowest percentage of sexually active women who were planning to use contraceptive methods at 17.3% (95% CI: 16.4, 18.2).

### Distribution of women with intention to utilise contraceptive methods by background characteristics and region

The results of a bivariate analysis of the relationship between each independent variable and the intention to utilise contraceptive method are presented in Table 2, which includes data from women in Africa, Asia, Latin America, and Eastern and Southern Europe. In all four regions, age of a woman was significantly associated with intent to use contraception. Intention to use contraception was highest among young women aged 15–24 years in Africa (54.5%), Asia (53.4%), and Latin America and the Caribbean (83.6%). However, in Eastern and Southern Europe, contraceptive use intention was highest among women aged 25–34 years (26.0%).

**Table 2.** Distribution of fecund females aged 15–49 years with intention to use contraception by background characteristics, DHS data.

Background characteristics	Africa (*N* = 285,662)	Asia (*N* = 357,019)	Latin America and the Caribbean (*N* = 34,798)	Eastern and Southern Europe (*N* = 20,113)
**Age**	***	***	***	***
15–24	54.5 [54.0,55.1]	53.4 [52.5,54.3]	83.6 [82.6,84.6]	18.8 [17.3,20.3]
25–34	48.6 [48.0,49.1]	46.1 [45.3,47.0]	76.6 [75.4,77.7]	26.0 [24.1,28.0]
35–49	27.1 [26.7,27.6]	18.9 [18.4,19.4]	40.7 [39.3,42.2]	10.0 [9.0,11.0]
**Residence**	***	***	***	***
Urban	44.7 [44.0,45.3]	41.8 [40.9,42.7]	70.4 [69.3,71.5]	15.7 [14.7,16.7]
Rural	42.8 [43.3,44.4]	35.8 [35.1,36.5]	65.6 [64.5,66.8]	19.7 [18.0,21.4]
**Education level**	***	***	***	***
None	32.7 [32.2,33.2]	26.9 [25.9,28.0]	42.5 [40.5,44.6]	29.2 [27.7,30.9]
Primary	47.7 [47.1,48.2]	41.4 [40.4,42.4]	65.5 [64.3,66.7]	14.1 [12.9,15.4]
Secondary	52.4 [51.8,53.0]	40.8 [40.0,41.6]	74.6 [73.4,75.9]	14.2 [12.8,15.7]
Higher	52.6 [51.1,54.0]	40.5 [39.3,41.7]	75.4 [73.3,77.3]	16.8 [14.4,19.5]
**Marital status**	***	***	***	***
Never married	56.2 [55.4,57.0]	59.7 [57.5,61.8]	77.7 [76.3,79.1]	2.5 [1.8,3.3]
Married	41.8 [41.4,42.2]	39.4 [38.8,40.0]	64.2 [63.2,65.3]	25.6 [24.1,27.1]
Formerly married	35.9 [35.2,36.7]	16.2 [15.2,17.2]	67.5 [66.0,69.1]	15.1 [12.8,17.8]
**Wealth status**	***	***	***	**
Poor	40.9 [40.4,41.5]	36 [35.3,36.8]	67.2 [66.0,68.3]	18.5 [17.2,20.0]
Middle	43.9 [43.1,44.6]	38.6 [37.6,39.7]	71.8 [70.2,73.4]	18.0 [16.2,19.9]
Rich	45.9 [45.3,46.4]	40 [39.2,40.9]	67.8 [66.3,69.2]	15.8 [14.6,17.1]
**Employment status**	Ns	***	***	***
Not working	43.6 [43.1,44.2]	38.1 [37.4,38.8]	71.6 [70.6,72.6]	19.1 [18.1,20.2]
Working	43.5 [43.0,43.9]	40 [39.1,40.8]	65.8 [63.7,66.0]	12.1 [10.8,13.7]
**Living children**	***	***	***	***
0	49.4 [48.7,50.1]	49.2 [48.2,50.2]	77.0 [75.7,78.3]	12.9 [11.9,14.0]
1–2	47.9 [47.4,48.4]	42.2 [41.5,43.0]	72.6 [71.5,73.7]	23.0 [21.2,24.8]
3–4	41.0 [40.5,41.6]	32.0 [31.1,32.9]	57.8 [55.6,60.0]	15.0 [13.4,16.8]
5+	34.1 [33.5,34.6]	23.1 [22.2,24.0]	41.7 [39.8,43.6]	23.9 [20.6,27.6]
**Number of under-five children**	***	***	***	***
0	37.0 [36.5,37.6]	33.1 [32.5,33.7]	63.7 [62.6,64.9]	12.7 [11.9,13.6]
1	46.3 [45.7,46.8]	43.2 [42.4,44.1]	73.3 [71.9,74.5]	23.8 [22.1,25.7]
2+	45.8 [45.2,46.3]	40.5 [39.5,41.5]	72.3 [70.5,73.9]	31.1 [27.7,34.7]
**Pregnancy loss**	***	*	***	Ns
No	43.7 [43.3,44.1]	38.5 [37.9,39.1]	70.0 [69.2,70.9]	16.9 [16.0,17.8]
Yes	42.4 [41.7,43.1]	37.4 [36.4,38.4]	60.6 [58.6,62.7]	18.7 [16.7,20.8]
**Fertility intention**	***	***	***	***
Have another child	46.3 [45.8,46.7]	48.3 [47.5,49.1]	74.8 [73.7,75.8]	42.2 [39.8,44.7]
No more	39.6 [39.1,40.1]	32.1 [31.4,32.8]	59.1 [57.7,60.4]	14.4 [13.3,15.7]
Undecided	33.7 [32.6,34.8]	26 [24.9,27.2]	74.3 [68.3,79.4]	21.7 [17.7,26.2]
**Distance to health facility**	***	**	***	***
Not a big problem	45.6 [45.2,46.1]	37.8 [37.1,38.5]	68.4 [67.0,69.7]	24.6 [22.5,26.9]
Big problem	41.8 [41.3,42.4]	36.1 [35.3,37.0]	63.9 [62.5,65.2]	32.1 [30.0,34.2]
**Ownership of radio**	***	Ns	*	***
No	41.9 [41.4,42.3]	36.2 [35.6,36.9]	66.9 [65.7,68.2]	15.2 [13.9,16.5]
Yes	44.6 [44.2,45.1]	35.3 [34.4,36.3]	68.7 [67.7,69.7]	18.3 [17.2,19.4]
**Exposure to FP messages**	***	***	***	Ns
No	38.8 [38.4,39.3]	36.0 [35.3,36.7]	64.4 [63.2,65.6]	16.8 [15.8,17.8]
Yes	51.3 [50.8,51.9]	41.2 [37.6,38.6]	73.7 [72.8,74.7]	18.6 [17.0,20.3]
**Total**	43.5 [43.1, 43.9]	38.1 [37.6,38.8]	68.0 [67.5,69.9]	17.3 [16.4,18.2]

***p < 0.001; **p < 0.01; *p < 0.05; Ns = non-significant.

Apart from Eastern and Southern Europe, living in urban places was linked to a high likelihood of intention to use contraception. The lowest prevalence of intention to use by residence was observed in Eastern and Southern Europe (15.7% in urban versus 19.7% in rural) compared to (70.4% in urban versus 65.6% in rural) for Latin America and the Caribbean.

Education was positively linked with women’s intent to utilise contraceptives in all these regions. Women who had no education and with a primary level of education had lower reports of contraceptive use intention in Africa (32.7%), Asia (26.9%), and Latin America and the Caribbean (42.5%). Women with tertiary education (52.6% in Africa), those with primary-level education (41.4% in Asia), and those with tertiary education (75.4% in Latin America and the Caribbean) had the highest rates of intention to use contraception.

The never-married women in all these regions, except for Eastern and Southern Europe, had higher rates of intention to utilise contraception methods. Wealth status was also significantly associated with contraception intention in all the regions. Women from poor households (40.9% in Africa, 36.0% in Asia, and 67.2% in Latin America and the Caribbean) had lower reports of contraceptive use intention when compared to those from middle and rich households. On the other hand, women from rich households (15.8%) in Eastern and Southern Europe had lower rates of contraceptive use intention (Table 2). Furthermore, women who had exposure to FP messages in Africa, Asia, and Latin America and the Caribbean had higher reports of intention to use contraceptive methods compared to their counterparts who had no exposure to FP messages.

### Determinants of contraceptive use intention

Multivariable-logistic-regression-adjusted odds ratios for the association between women’s background characteristics and future desire to utilise contraception in all four regions are shown in Table 3. Level of education, work position, parity, number of under-five children, pregnancy loss, fertility desire, distance to health facility, radio ownership, and exposure to FP messaging were all substantially linked to higher likelihoods of planning to use contraception globally. Results from all regions indicate that a woman’s likelihood of intending to utilise contraception decreased as her age increased. Females aged 35–49 years had lower odds of planning to adopt contraception in comparison to those in the age range of 15–19 years in Africa (aOR = 0.27; 95% CI: 0.26–0.28), Asia (aOR = 0.20; 95% CI: 0.18–0.22), Latin America (aOR = 0.13; 95% CI: 0.11–0.16), and Eastern and Southern Europe (aOR = 0.15; 95% CI: 0.12–0.20).

**Table 3.** Adjusted odds ratios of the multivariable binary logistic regression of the association between background variables and contraceptive use intention among sexually active women, DHS data.

Background characteristics	Africa (*N* = 285,660)	Asia (*N* = 357,019)	Latin America and the Caribbean (*N* = 34,798)	Eastern and Southern Europe (*N* = 20,113)	All regions
	aOR (95% CI)	aOR (95% CI)	aOR (95% CI)	aOR (95% CI)	aOR (95% CI)
**Age**					
15–24	1	1	1	1	1
25–34	0.74*** (0.71–0.76)	0.80*** (0.76–0.85)	0.60*** (0.53–0.68)	0.69** (0.55–0.86)	0.75***(0.73–0.76)
35–49	0.27*** (0.26–0.28)	0.20*** (0.18–0.22)	0.13*** (0.11–0.16)	0.15*** (0.12–0.20)	0.24***(0.24–0.25)
**Residence**					
Urban	1	1	1	1	1
Rural	1.23*** (1.17–1.27)	0.86*** (0.79–0.92)	0.93 (0.81–1.07)^**Ns**^	0.94 (0.72–1.23)^**Ns**^	1.03 (1.00–1.07)^**Ns**^
**Education level**					
None	1	1	1	1	1
Primary	1.66*** (1.61–1.71)	1.87*** (1.73–2.03)	1.72*** (1.51–1.97)	0.27*** (0.21–0.33)	1.72***(1.68–1.77)
Secondary	1.93*** (1.86–2.00)	1.40*** (1.29–1.51)	2.01*** (1.71–2.37)	0.13*** (0.10–0.16)	1.68***(1.62–1.72)
Higher.	2.15*** (2.01–2.31)	1.21*** (1.10–1.34)	2.69*** (2.16–3.35)	0.10*** (0.07–0.14)	1.71***(1.63–1.80)
**Marital status**					
Never married	1	1	1	1	1
Married	0.76*** (0.73–0.81)	0.85 (0.66–0.09)^Ns^	1.07* (0.98–1.22)	0.86 (0.41–1.78)^Ns^	0.76***(0.73–0.79)
Formerly married	0.68*** (0.65–0.72)	0.34*** (0.26–0.45)	1.51*** (1.28–1.77)	0.42* (0.19–0.91)	0.70***(0.66–0.73)
**Wealth status**					
Poor	1	1	1	1	1
Middle	1.04* (1.00–1.07)	0.96 (0.90–1.02)^**Ns**^	1.14 (0.98–1.32)^**Ns**^	1.30* (0.99–1.71)	1.01(0.98–1.04)^**Ns**^
Rich	1.06** (1.01–1.11)	0.99 (0.92–1.05)^**Ns**^	0.76** (0.64–0.89)	1.55** (1.19–2.03)	0.99(0.96–1.03)^**Ns**^
**Employment status**					
Not working	1	1	1	1	1
Working	1.15*** (1.12–1.19)	1.44*** (1.36–1.52)	1.01 (0.92–1.10)^**Ns**^	1.12 (0.90–1.10)^**Ns**^	1.21***(1.18–1.24)
**Living children**					
**0**	1	1	1	1	1
**1–2**	1.13*** (1.08–1.18)	0.99 (0.92–1.07)^**Ns**^	1.24** (1.07–1.44)	0.84 (0.66–1.06)^**Ns**^	1.04* (1.01–1.08)
**3–4**	1.28*** (1.22–1.35)	0.83*** (0.75–0.91)	1.34* (1.07–1.67)	0.88 (0.65–1.20)^**Ns**^	1.08***(1.04–1.13)
5+	1.41*** (1.33–1.49)	0.78*** (0.69–0.88)	1.16 (0.92–1.46)^**Ns**^	0.79 (0.55–1.13)^**Ns**^	1.23***(1.16–1.29)
**Number of under-five children**					
0	1	1	1	1	1
1	1.28*** (1.22–1.35)	1.35*** (1.27–1.44)	1.34*** (1.19–1.51)	1.58*** (1.29–1.92)	1.42***(1.38–1.46)
2+	1.41*** (1.33–1.49)	1.17*** (1.09–1.26)	1.28** (1.10–1.49)	2.28*** (1.72–3.01)	1.36***(1.32–1.40)
**Pregnancy loss**					
No	1	1	1	1	1
Yes	1.10 *** (1.06–1.13)	1.16*** (1.09–1.23)	0.88* (0.78–1.00)	1.02 (0.84–1.23)^**Ns**^	1.06***(1.03–1.09)
**Fertility preference**					
Have another child	**1**	1	1	1	1
No more	1.25*** (1.21–1.29)	1.36*** (1.28–1.46)	0.91 (0.79–1.04)^**Ns**^	0.52*** (0.44–0.62)	1.22***(1.18–1.25)
Undecided	0.73*** (0.69–0.77)	0.52*** (0.48–0.57)	1.28 (0.82–2.00)^**Ns**^	0.41*** (0.30–0.58)	0.56***(0.56–0.61)
**Distance to health facility**					
Not a big problem	1	1	1	1	1
Big problem	0.91*** (0.89–0.94)	1.00 (0.94–1.06)^Ns^	0.89*(0.81–0.98)	1.05 (0.89–1.24)^**Ns**^	0.95***(0.93–0.98)
**Ownership of radio**					
No	1	1	1	1	1
Yes	0.98 (0.96–1.01)^**Ns**^	0.86*** (0.81–0.91)	1.07 (0.97–1.69)^**Ns**^	1.72*** (1.47–2.02)	1.05***(1.03–1.07)
**Exposure to media FP messages**					
No	1	1	1	1	1
Yes	1.60*** (1.56–1.65)	1.39*** (1.31–1.48)	1.54*** (1.40–1.69)	1.87*** (1.57–2.23)	1.51***(1.48–1.55)

***p < 0.001; **p < 0.01; *p < 0.05; Ns = non-significant.

Women in rural parts of Africa had a higher likelihood of intending to use contraception (aOR = 1.23; 95% CI: 1.17–1.23). In contrast, Asian women living in rural regions had a lower likelihood of intending to use contraception than those residing in urban areas (aOR = 0.86; 95% CI: 0.79–0.92). As expected, higher levels of education among women were strongly linked to higher likelihoods of planning to use contraception across Latin America, Asia, and Africa. In comparison to women without education, women with more than a secondary education had higher odds of planning to use contraception in future than those with primary level of education in Africa (aOR = 2.15; 95% CI: 2.01–2.31), Asia (aOR = 1.21; 95% CI: 1.10–1.34), and Latin America (aOR = 2.69; 95% CI: 2.16–3.35). In contrast, women with more than secondary level of education in Eastern and Southern Europe had lower odds of intending to utilise contraceptives than their counterparts who had no formal education (aOR = 0.10, 95% CI: 0.07–0.14).

Women in Africa and Eastern and Southern Europe who lived in wealthy households had higher odds of planning to utilise contraceptives than those from poor households (aOR = 1.06; 95% CI: 1.01–1.11) and (aOR = 1.55; 95% CI: 1.19–2.03), respectively. Similarly, women who were working in Africa (aOR = 1.15; 95% CI: 1.12–1.19) and Asia (aOR = 1.44; 95% CI: 1.36–1.52) had higher odds of intending to use contraception than those who were not working.

In each of the four locations, there was a correlation between the number of under-five children present in the household and the intention to use contraception. Women from households that had two or more children in Africa (aOR = 1.41; 95% CI: 1.33–1.49), Asia (aOR = 1.17; 95% CI: 1.09–1.23), Latin America and the Caribbean (aOR = 1.28; 95% CI: 1.10–1.49), and Eastern and Southern Europe (aOR = 2.28, 1.72–3.01) were more likely to intend to use contraception than women from households with no children under the age of 5. Women who experienced a pregnancy loss in Africa (aOR = 1.10; 95% CI: 1.06–1.13) and Asia (aOR = 1.16; 95% CI: 1.09–1.23) were more willing than other women to utilise birth prevention methods later. Exposure to FP messages was associated with high likelihood of wanting to take contraception across all regions.

## Discussions

The aim of the current study was to explore patterns and predictors of intention to use contraceptives among sexually active fecund females in low- and middle-income countries. The study included countries from Africa, Asia, Eastern and Southern Europe, and Latin America. This study found that overall, 42.8% (95% CI: 42.5, 43.1) of fecund sexually active women had the intention to use contraceptives methods, with developing countries from Eastern and Southern Europe having the lowest pooled prevalence of contraceptive intention of 17.3% (95% CI: 16.4, 18.2). The highest percentage of those intending to utilise contraceptive methods was noted in Latin America and the Caribbean, 68.0% (95% CI: 67.5, 69.9). In terms of country-level analysis, Albania, 11.3% (95% CI: 9.7, 13.1) had the lowest proportion of females who showed willingness to utilise contraceptive methods, while Honduras had the highest proportion at 80.6% (95% CI: 79.5, 81.2). The disparities in the sociocultural and demographic backgrounds across the countries and regions taken into consideration in this study may help to explain the discrepancies in contraceptive use intention behaviour.

According to the results obtained, older women were less likely than younger ones in every location to plan to take contraception. This supports results from prior studies conducted in Ethiopia [40,41], Jordan [42], Malawi [43], Pakistan [17], Mozambique [44], and Uganda [45]. This finding has a significant implication for designing of programmes promoting contraception use among older women. A likely explanation for these findings is that young women were more inclined to delay having children, because they were involved in other activities, such as pursuing their educational careers. This suggests that young women may be more likely to plan to take birth control [46–48]. In order to meet reproductive goals, as a woman gets old, her likelihood of conception may decrease [40,42,49].

The intention of sexually active women to adopt contraceptive methods was found to correlate with education. At a global level, compared to women without formal education, women with formal education have a higher probability of intending to utilise contraceptives in the future. The finding of this study is consistent with studies conducted across different countries and regions [41,42,45,49,50]. This conclusion could be explained by the fact that females who have formal education have a higher likelihood of being exposed to messages about contraceptive use benefits through the media and other sources of information. This contributes to an increased awareness of and access to contraceptive methods, and their ability to comprehend the health benefits of the contraception in lowering fertility, unintended pregnancy, unsafe abortions, and other issues affecting mothers and children [19,39,51–54].

This study found that women who were residing in rural settings in Africa exhibited higher intention to utilise contraceptive methods in comparison to their counterparts who were living in urban areas. This finding has important implications for the design of contraception promotion interventions targeting women in marginalised communities, such as rural areas. Some studies conducted in Sub-Saharan Africa counties [40,49] also confirm the conclusion that women who were residing in rural areas were more likely to have the intention to use contraceptives, as compared to those residing in urban settings. This is an unexpected finding. Further studies will be essential to probe the reasons why women who live in urban areas would have a low intention of utilising contraceptive methods.

Women’s empowerment is an important element for enhancing maternal health [55–58]. According to the results of our study, women in employment had a higher likelihood of planning to utilise contraceptive methods in comparison to their counterparts who were not employed. This finding is consistent with prior studies done in certain Sub-Saharan African countries [4,46,49,59,60] as well as Asian countries [61–64]. Furthermore, being employed enables women to become more independent, and have autonomy over their own health, which helps them make better health-related decisions [55,56,65]. It is noteworthy that employed women may not wish to become pregnant, as this may interfere with their ability to work.

## Study limitations

As in any other empirical research, this study has limitations. Even though the current study produced several significant findings, the cross-sectional method of the DHS prevents it from demonstrating the causal relationship between the outcome of interest and the independent correlates. Furthermore, our study was unable to examine association of qualitative variables such as religious beliefs, norms, and sociocultural factors with the intention of contraceptive usage among women, because the DHS data did not include qualitative data. Further, the study only included four countries from Eastern and Southern Europe, because most countries in the region have outdated DHS data, making the number of countries small to represent the entire region. Despite the limitations, the study’s major strength was that it used nationally representative cross-sectional data collected by the DHS programme from 69 countries globally. Therefore, the study findings could be inferred to represent the entire population of sexually active women who are in the reproductive age in each country. In addition, the DHS used validated and standardised data collection instruments in its appraisals of datasets, along with its large sample sizes. Furthermore, the well-designed procedures, such as the training field enumerators and employing well-tested methods for data collection, improved the quality of the data collected to inform the strengthening of health policy and programme design aimed at improving contraception uptake among women.

## Conclusion

According to this study, the percentage of sexually active women in many developing countries who have the intention to utilise contraceptive methods is low. This has the potential to contribute to high rates of unplanned pregnancies. Level of education, age of a woman, employment status, fertility preference, number of living children, and exposure to media were identified as the main predictors of contraceptive use intention at the global level. Regional differences in the prevalence of intention to utilise contraceptives suggest the need for concerted efforts to address inequalities in access to maternal health promotion services. Therefore, health policy-makers and FP implementing agencies in the various countries considered in this current study ought to ensure that barriers to women’s reproductive health autonomy are addressed. This can be done through intensifying access to education and strengthening of women’s empowerment initiatives for women in marginalised communities. To increase contraceptive uptake, it will be important to expand access to maternal health promotion messages broadcast via mass-media channels that target both young and older women. It is imperative to conduct further research in order to explain why women living in urban areas have low intention to use contraceptive methods.

## Paper context

Utilisation of contraceptives among women is widely recognised as a global public health strategy for reducing maternal mortality. While extensive research exists on this subject, little global attention has been paid to improve understanding of the drivers of women’s contraceptive use intention. This study helps fill that gap by providing insights into the characteristics of women who intend to use contraceptives. This information serves to inform future design of family planning interventions.
